# Analyzing Differences in Viral Dynamics Between Vaccinated and Unvaccinated RSV Patients

**DOI:** 10.3390/epidemiologia6020016

**Published:** 2025-04-01

**Authors:** Arjan Suri, Sahaj Satani, Hana M. Dobrovolny

**Affiliations:** Department of Physics & Astronomy, Texas Christian University, Fort Worth, TX 76129, USA

**Keywords:** respiratory syncytial virus, vaccine, viral clearance, mathematical model

## Abstract

**Background:** Respiratory syncytial virus (RSV) is a common respiratory virus that can cause serious illness in infants and the elderly. Vaccines for RSV have recently been introduced and have been shown to reduce the severity of the disease. However, there has been limited examination of how viral dynamics differ between vaccinated and unvaccinated individuals. **Methods:** Here, we use data from the MVA-BN-RSV Phase II vaccine study to quantify the dynamical differences between vaccinated and unvaccinated patients challenged with RSV. We use an ordinary differential equation model of within host viral dynamics to fit viral load data. **Results:** We find statistically significant differences in viral clearance rate and basic reproduction number. We also find that vaccinated patients experience a higher response variance than the placebo group. **Conclusions:** While the differences in viral clearance and basic reproduction number are promising, the high variability in response to the vaccine could leave many vaccinated patients without adequate protection.

## 1. Introduction

Respiratory syncytial virus (RSV) causes considerable respiratory disease, mostly in young infants and the elderly [1]. The burden of RSV in older adults causes 60,000–160,000 hospitalizations and 6000–10,000 deaths per year in the United States, similar to the impact of influenza [2]. It also tends to develop into bronchiolitis, pneumonia, asthma, and can cause worsening of chronic obstructive pulmonary disease (COPD) or exacerbation of congestive heart failure for the older population [3]. In addition, RSV is the most common cause of lower respiratory tract infection in young children [4]. Highly infectious, RSV infects 70% of children in their first year of life, and almost all children by 2 years of age [5]. RSV infection leads to 3.6 million estimated pediatric hospital admissions and 101,400 child deaths per year globally [6]. While RSV disproportionately causes serious illness in the younger and older populations [7], it is still a leading cause of acute respiratory tract infection amongst all humans [8]. Therefore, it bears a considerable burden for all age groups, particularly for immunocompromised patients or patients with other comorbidities [9,10,11].

**Figure 1:**
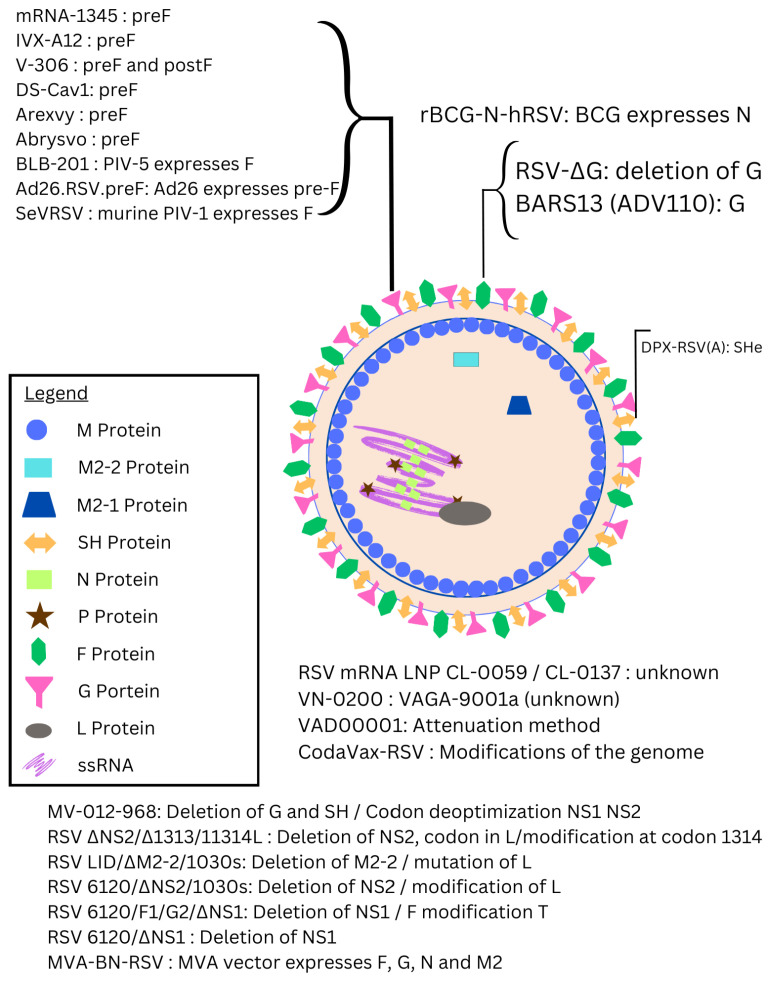
Structure of the RSV virion and categorization of vaccine candidates [3]. Vaccines often target the F and G proteins on the surface of the RSV virion.

RSV is an enveloped, negative-sense, single-stranded RNA virus belonging to the *Paramyxoviridae* family that causes seasonal infections in a bimodal age distribution, with subtypes RSV Type A (RSV-A) and RSV Type B (RSV-B) causing infection throughout the season, one of two prevailing each year [12]. RSV consists of eleven structural and non-structural proteins, with the fusion (F), attachment (G), and small hydrophobic (SH) proteins located on its outer lipid membrane. The F protein is essential for the fusion of the virus with host cell membranes and attachment [13], a process primarily driven by the glycosylated G protein [14], which varies across RSV strains. The SH protein aids in viral replication and delaying apoptosis, with its removal leading to viral weakening [15]. Additional proteins, including M2-1, M2-2, and NS1/NS2, regulate transcription and inhibit immune responses, while the Matrix (M) protein and RNA polymerase complex support viral replication [16]. A number of these viral proteins have been targets for vaccine development (see Figure 1).

In dealing with RSV, therapeutic interventions are limited; many patients receive supportive care such as providing adequate hydration, decongestion, and oxygen supply [17], and the antiviral ribavirin appears to have some clinical efficacy in treating RSV [18]. Prevention strategies include measures to avoid exposure and passive/active immunization [19]. So, since the discovery of RSV in 1956, many individuals and organizations have created vaccines to address the burden of its morbidity [3]. There are four types of vaccines that have been developed and tested against RSV:

- mRNA Vaccines: Although mRNA vaccines rose to prominence with COVID-19, their application in RSV has been more recent, largely inspired by the success seen during the pandemic [20]. Efforts to develop mRNA vaccines for RSV are underway [21,22], with one mRNA vaccine already having received FDA approval [23]. Other mRNA vaccines are still being investigated, with the aim of achieving rapid, scalable production and targeted immune responses for populations at high risk, such as infants and older adults.
- Live-attenuated RSV vaccines have been in development for decades. However, early versions had setbacks, such as enhanced respiratory disease (ERD) in some young children, prompting significant adjustments [24]. Today, improved live-attenuated RSV vaccines are being tested, particularly those that are safer for RSV-naïve infants [25,26].
- Subunit/Virus-Like Particle (VLP)-Based Vaccines: Subunit vaccines, which consist of purified pathogen fragments, gained traction after the approval of the hepatitis B vaccine in 1986 [27]. Subunit vaccines targeting the RSV F protein have long been a focus due to the protein’s role in viral fusion [28,29]. Virus-like particles represent a recent advancement, resembling the virus structure but lacking the ability to replicate, making them safer for vulnerable populations [30].
- Recombinant Viral-Vector-Based Vaccines: The use of viral vectors in vaccines began in 1972 [31], with recent developments harnessing modified viruses to deliver genetic information and produce target proteins. This strategy provides flexibility in targeting the RSV proteins most critical for immunity, like F and G, and has shown promise for long-term efficacy in clinical studies [32].

In the last couple of years, a number of vaccines have been approved for use in patients over 60 years of age [33,34] and one is approved for maternal use to protect newborns [35]. While the efficacy of these vaccines is fairly high in the elderly population, with an estimated 90% protection from lower respiratory tract infection [36], the maternal vaccine is not quite as effective [35]. Thus more work is needed to improve our understanding of patient response to vaccination. A recent potential RSV vaccine candidate, MVA-BN-RSV [37,38], provides an interesting case study. The vaccine, a nonreplicating, modified vaccinia Ankara (MVA) virus encoding both the F and G proteins, showed promising results in a phase IIa trial [37], but failed to meet its success criteria in the phase III trial [38]. In the phase III trial, the vaccine efficacy was 42.9% against RSV-associated lower respiratory tract disease (LRTD) with ≥3 symptoms, 59.0% against LRTD with ≥2 symptoms, and 48.8% against acute respiratory distress (ARD). Of particular interest are the 95% confidence intervals found in the study for the estimates of efficacy. The 95% CI is −16.1–71.9 for LRTD with ≥3 symptoms; 34.7–74.3 for LRTD with ≥2; and 25.8–64.7 for ARD. The range of possible outcomes after vaccination with MVA-BN-RSV appears to be broad, so while the vaccine can provide good protection for some, others receive little protection from the vaccine.

Mathematical models have been revealed as a powerful tool to improve our understanding of viral infection dynamics [39,40,41], and to help estimate values of different quantities relevant to the replication process [42,43]. Mathematical models have been used to study a number of aspects of RSV including estimating parameters that characterize the replication cycle [44,45,46,47]. Models have also been used to examine the effect of the immune response [48], temperature and pH [49], treatment [50,51], syncytia [52], defective viral genomes [53], age [54], and spatial motion [55] on the time course of RSV infections. Since approved vaccines are a recent development for RSV, mathematical models have not yet been used to study the potential effects of vaccines on within host dynamics. Using mathematical modeling to estimate parameters characterizing the viral replication cycle could be a useful tool in helping researchers understand the effect of vaccination on within host viral replication.

In this paper, we analyze the differences in viral load dynamics between vaccinated and unvaccinated patients using data from the MVA-BN-RSV Phase II vaccine trial, with the aim of providing insight into why the vaccine subsequently failed in the phase III trial. We employ a within host ordinary differential equation (ODE) model to fit the infectious viral load and RNA viral load data, allowing us to quantitatively assess the differences in viral dynamics between the vaccinated and placebo groups. Consistent with expectations, we find that the vaccinated group has a higher viral clearance rate and lower basic reproduction number. However, our analysis also reveals that vaccinated patients exhibit a higher response variance compared to unvaccinated individuals, suggesting variability in the immune response induced by the MVA-BN-RSV vaccine. Variability in the immune response could explain the large confidence intervals in vaccine effectiveness observed in the phase III trial.

## 2. Materials and Methods

### 2.1. Experimental Data

A study performed by Jordan et al. assesses the effectiveness of MVA-BN-RSV, a novel poxvirus-vectored vaccine encoding internal and external RSV proteins [37]. In phase IIa of their trial, participants aged 18 to 50 years were given MVA-BN-RSV or treated with a placebo in a double-blind process. Participants were challenged with RSV-A Memphis 37b 4 weeks after vaccination and viral load samples were taken for 12 days. Viral load was measured twice daily from nasal washes using both qRT-PCR and plaque assay.

### 2.2. Mathematical Model

We use a simple mathematical model of within host viral dynamics that includes both infectious and non-infectious virus [56],

(1) $$\begin{array}{cc} \frac{dT}{dt} & =-\beta TV_{\inf}\\ \frac{dI}{dt} & =\beta TV_{\inf}-\delta I\\ \frac{dV_{\inf}}{dt} & =pI-c_{\inf}V_{\inf}\\ \frac{dV_{\mathrm{RNA}}}{dt} & =\rho pI-c_{\mathrm{RNA}}V_{\mathrm{RNA}}. \end{array}$$

In this model, uninfected target cells (*T*) are being infected by the virus with infection rate β (/([$V_{\inf}$ ]·[time])). Once target cells are infected (*I*), they produce infectious virus ($V_{\inf}$) at a rate *p* ([$V_{\inf}$ ]/([cell]·[time])) and viral genomes ($V_{\mathrm{RNA}}$) at rate ρp ([$V_{\mathrm{RNA}}$ ]/([cell]·[time])). Infected cells die at a rate δ (1/[time]), where δ is the infected cell decay rate. Infectious virus decays at a rate $c_{\inf}$ (1/[time]), while viral genomes decay at a rate $c_{\mathrm{RNA}}$ (1/[time]). We do not explicitly include an immune response in order to minimize the number of free parameters. We assume that the effect of the immune response will appear as differences in values of parameter estimates between the vaccinated and unvaccinated groups.

### 2.3. Fitting the Model to Data

We use WebPlotDigitizer (https://automeris.io, accessed on 28 May 2024) to extract mean viral load data from Figure 2A,B of [37]. We used model Equation (1) to fit the data. We assumed the following initial conditions: T(0)=1, I(0)=0, $V_{\inf}$ (0) = 0.01 pfu/mL, and $V_{\mathrm{RNA}}$ (0) = 0.1 copies/mL. Unfortunately, the amount of virus in the initial inoculum is not given in the original manuscript, so we chose a value below the threshold of detection (0 $\log_{10}$(pfu/mL) and 0 $\log_{10}$(copies/mL)) while allowing for $V_{\mathrm{RNA}}$ to be roughly 10 times larger than $V_{\inf}$. We found the best parameter values through minimization of the Sum of Squared Residuals (SSR),

$$SSR=\sum_{i=1}^{D}{\left(f\left(x_{i}\right)-y_{i}\right)}^{2},$$

where $f(x_{i})$ are the estimated model values and $y_{i}$ are the experimental data points. Note that both $V_{\inf}$ and $V_{\mathrm{RNA}}$ are included in the calculation of the *SSR*.

We used scipy’s minimize function with the Nelder-Mead algorithm. The Nelder-Mead algorithm, also known as the “downhill simplex” technique [57], is particularly suited for this context as it does not require derivative information, making it good for noisy, complex, or non-differentiable functions, which provides a framework for minimizing the error between predicted and observed viral loads. The free parameters we estimated include β, *p*, ρ, δ, $c_{\inf}$, and $c_{\mathrm{RNA}}$. We also used the estimated parameter values to estimate two more quantities that characterize spread of the virus within a host. The first is the basic reproduction number, $R_{0}$, which gives the number of secondary infections caused by a single infected cell. It is given by

$$R_{0}=\frac{\beta p}{c\delta }.$$

The second quantity is the infecting time, given by

$$t_{\inf}=\sqrt{\frac{2}{p\beta }},$$

that represents the average time it takes for a virion to be released from one cell and infect the next.

Once we found the best-fit parameters, we used bootstrapping to estimate the variability and confidence intervals of model parameters. Bootstrapping is a resampling technique that involves repeatedly drawing samples from the original data and re-estimating parameters for each sample [58]. This allows us to approximate the sampling distribution of the parameters without assuming a specific distribution.

We used two statistical tests to assess differences in parameter estimates between the vaccinated and unvaccinated groups. The Wilcoxon Rank-Sum test was used to assess differences in median values of the parameters. We used the Levene Test to determine differences in variance. In order to avoid oversampling, we ran each test 100 times with 20 random samples from each distribution [53]. We calculated the average p value and consider p values less than 0.05 as statistically significant.

## 3. Results

### 3.1. Model Fit to Data

**Figure 2:**
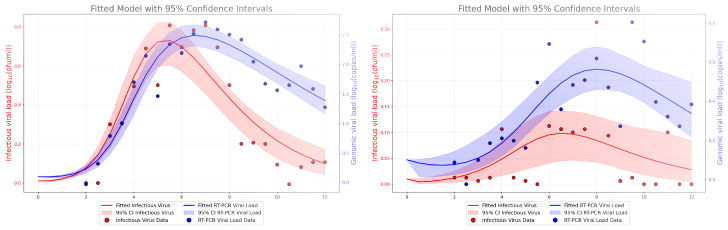
Clinical viral load measurements and model best fits for the unvaccinated (**left**) and vaccinated (**right**) groups. Blue lines indicate the time course of the non-infectious virus while the red line shows the time course of infectious virus. The shaded region indicates the 95% CI of the best fit model prediction.

We use an ordinary differential equation (ODE) model (Equation (1)) to replicate viral dynamics in both vaccinated and unvaccinated groups. We fit the model simultaneously to both infectious and genomic viral measurements as described in the methods. Results are shown in Figure 2. Best fit parameter values are given in Table 1.

The model fits both groups fairly well, although there is clearly more variability in the measurements taken from the vaccinated group. Both the genomic and infectious viral loads in the unvaccinated group show a clear rise and fall and both reach much higher levels than the vaccinated group. The infectious viral load in the vaccinated group barely rises above the threshhold of detection. Despite the clear differences in viral loads, viral production rate, both infectious and non-infectious, is similar for the two groups, but the infection rate for the unvaccinated group is about half the infection rate for the vaccinated group. There are more notable differences in the clearance rates between the two groups. The death rate of infectious cells is slightly higher in the vaccinated group while the viral clearance rates, both infectious and non-infectious, are about twice as fast in the vaccinated group than in the unvaccinated group.

We also calculated two additional parameters that characterize viral infections, the basic reproduction number, $R_{0}$, and the infecting time, $t_{\inf}$, (see methods for equations). The infecting time is slightly lower in the vaccinated group as compared to the unvaccinated group, although the difference is not statistically significant (see Section 3.3). The basic reproduction number gives the number of secondary infections caused by a single infected cell and is a measure of how quickly the virus replicates within the host. $R_{0}$ for the unvaccinated group is 13.8, while $R_{0}$ for the vaccinated group is 4.05 suggesting slower spread of the virus in vaccinated patients.

### 3.2. Assessment of Identifiability

**Figure 3:**
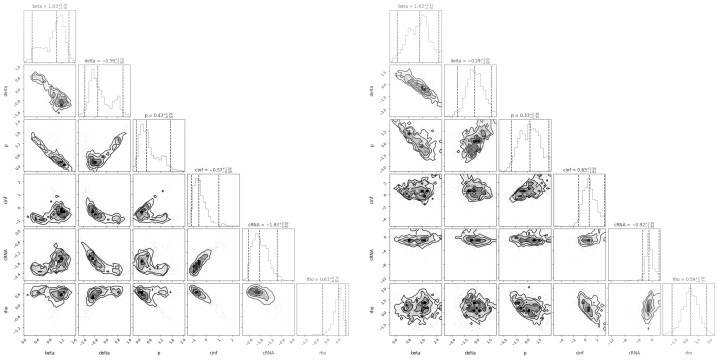
Correlation plots and histograms for unvaccinated (**left**) and vaccinated (**right**) groups. Figures off the diagonal show one parameter plotted against another with the parameters in the following order (top to bottom and left to right): β, δ, *p*, $c_{\inf}$, $c_{\mathrm{RNA}}$, ρ. The diagonal figures show the distributions for each parameter.

In an effort to assess how well we can identify the parameter values, we examined the correlation plots for the estimated parameters. The corner plots presented in Figure 3 show the distribution and correlation structure of model parameters for the unvaccinated (left) and vaccinated (right) groups. Each off-diagonal plot displays the pairwise relationships between parameters, with histograms along the diagonal representing individual parameter distributions.

The unvaccinated group exhibits a more elongated, directional structure in many of the parameter pairwise distributions. This elongation indicates stronger correlations between certain parameters. For example, parameters like β and δ (infection and cell death rates) display a pronounced linear relationship, implying a more consistent pattern of response within the unvaccinated group. In contrast, the vaccinated group shows more circular distributions in the pairwise comparisons. This rounder shape suggests weaker correlations and greater variability in the relationships among parameters, indicating a more dispersed, less predictable pattern of response. The vaccinated individuals display higher parameter variability, as seen in the wider spread of the histograms and reduced correlation in parameter relationships. The increased variability in parameter estimates for the vaccinated group could suggest that the vaccine affects the infection dynamics in a more individualized manner. This could imply that vaccinated patients respond more variably to infection, potentially due to differences in baseline health conditions, or varying levels of immune response efficacy.

### 3.3. Parameter Comparison

**Figure 4:**
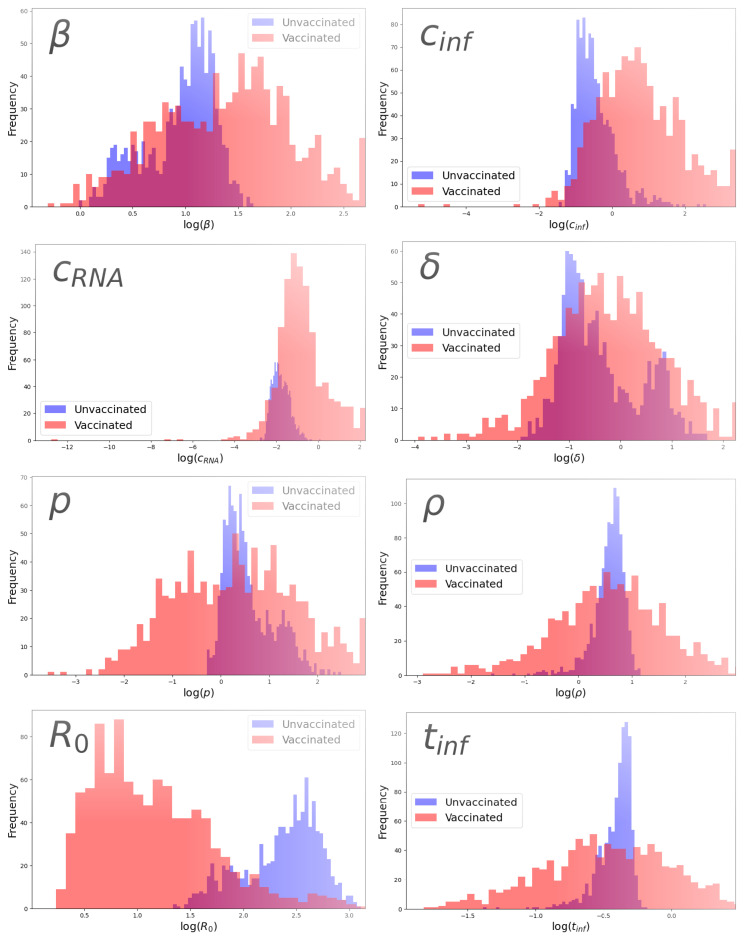
Comparison of parameter distributions for unvaccinated (blue) and vaccinated (red) groups for β (**top left**), $c_{\inf}$ (**top right**), $c_{\mathrm{RNA}}$ (**2nd row left**), δ (**2nd row right**), *p* (**3rd row left**), ρ (**3rd row right**), $R_{0}$ (**bottom left**), and $t_{\inf}$ (**bottom right**).

We noted some differences in the estimated parameter values for the vaccinated and unvaccinated groups. Figure 4 shows the parameter distributions for each parameter for the unvaccinated (blue) and vaccinated (red) groups. We note that most of the parameter distributions overlap to some extent, but there seems to be a difference in the spread of the distributions, with the vaccinated group generally having broader distributions than the unvaccinated group. For this reason, we perform two statistical tests: the Wilcoxon rank sum test compares the median values of the distributions, and the Levene test compares the variance of the distributions. Table 2 presents average *p*-values for both the Wilcoxon rank sum test and the Levene test for estimated viral parameters when comparing vaccinated and unvaccinated groups. Statistically significant *p*-values (*p* < 0.05) are in bold.

Both infectious and non-infectious viral clearance rates differed significantly between the vaccinated and unvaccinated groups, as did differences in the basic reproductive number. These indicate that the vaccine is generally having the desired effect—virus is cleared more rapidly in vaccinated patients and the infection spreads less efficiently. There were also statistically different variances in some of the parameters between the two groups. The infection rate had a bigger spread in the vaccinated group than in the unvaccinated group as did the infecting time. These two parameters characterize how easily the virus goes from one cell to another, suggesting that there is wide variability in how well the vaccine inhibits viral replication. The viral clearance rates also show more variability in vaccinated patients, which could be a more direct reflection of the effect of the vaccine in different individuals.

## 4. Discussion

This study highlights differences in viral dynamics between vaccinated and unvaccinated RSV patients. We found statistically significant differences in the viral clearance rates, $c_{\inf}$ and $c_{\mathrm{RNA}}$, as well as statistically significant differences in the basic reproduction number between vaccinated and unvaccinated groups. The increased viral clearance rate in vaccinated patients is likely responsible for the observed reduction in viral loads over the duration of the infection. Increased viral clearance is most likely due to an increased antibody response, since antibodies directly neutralize virus, effectively clearing them from the infection process. This suggests that overall, the vaccine is effective in enhancing viral clearance and reducing the virus’ ability to spread within the host. We also found, however, that many of the parameter estimates showed greater variability in vaccinated patients than in unvaccinated patients, suggesting a heterogeneous immune response among vaccinated participants.

The variability in vaccinated parameter estimates in this study arises from the large amount of scatter in the median viral load measurements of vaccinated patients. A better assessment of the variability in vaccine response would be to fit the model to viral titer curves from each individual patient. Previous work comparing parameter estimates from individual fits to parameter estimates from fits to median data found that the two methods resulted in slightly different parameter estimates [59]. There are a number of known factors that could contribute to the variability of the vaccine response. For example, the male and female immune response to RSV are known to differ [60]. BMI and other comorbidities can also play a role in the effectiveness of the immune response elicited by vaccination [61]. Finally, age can also affect the immune response to vaccination [62,63]. While the use of healthy adults is meant to minimize some of these factors, it does not eliminate all of them. Since Jordan et al. present only the median viral load, we cannot disentangle the effect of these different contributing factors.

The variability in vaccinated parameter estimates also leads to larger confidence intervals, leading to lower practical identifiability for these parameters. There are practical limitations for improving parameter identifiability in this case. To improve identifiability, we typically need to make measurements of additional variables [56,64,65]. The additional variables that could be measured for this model are target cells or infected cells, but it is not possible to measure those in patients. Another option is to extend the model to include other quantities that could be measured, such as antibodies [56,64]. Unfortunately, including more equations means that there are more parameters that need to be identified, so it is not immediately clear that this will improve identifiability of the original parameters. A final strategy is to measure some of the parameters using data from different experiments, such as monitoring viral decay to determine $c_{\inf}$ or $c_{\mathrm{RNA}}$ independently [47,66].

Another limitation of the data is that the study is performed in healthy adults. RSV vaccines are meant to protect those at higher risk of developing severe disease, including the elderly and infant populations [4,67,68]. RSV dynamics are known to be different in these populations in the absence of vaccination [46]. There is also known variability in the immune response to RSV infection in pediatric patients [69] that might also result in varied immune responses to an RSV vaccine in this population. Future studies should focus on longitudinal data collection so that mathematical models can be used to estimate parameter values, which will help quantitatively characterize differences in vaccine response between different groups.

The model used in this study is fairly simple. In particular, it does not include an explicit immune response, which is needed to properly model the effect of a vaccine. Models describing the effect of vaccines have been used to study the immune response to vaccination [70], helping researchers understand how strong a dose might be needed [71,72] and how many doses might be needed [72,73], although a mathematical model of response to RSV specifically has not yet been developed. Developing such a model would require detailed measurements of the immune response to vaccination and during the subsequent challenge infection. While some immune measurements were taken in this study, they were taken at only a few time points and were measured from serum samples, so did not represent the immune response at the site of the infection, making it difficult to use these measurements to validate a model with an explicit immune response. A comprehensive model of RSV vaccine response could help us understand why there appears to be such variability in the effectiveness of the MVA-BN-RSV vaccine. Future work should consider these elements, as they may be necessary to capture the more nuanced effects of RSV vaccination, beyond what our model currently provides.

## Figures and Tables

**Table 1:** Best fit parameter values and 95% Confidence Intervals for vaccinated and unvaccinated groups.

Parameter	Group	Parameter Value	95% CI
β (pfu/mL·d)^−1^	Unvaccinated	2.80	[1.29, 4.17]
	Vaccinated	4.18	[1.14, 12.7]
δ (/d)	Unvaccinated	0.554	[0.228, 3.24]
	Vaccinated	0.748	[0.0956, 5.59]
*p* (pfu/mL·d^−1^)	Unvaccinated	1.54	[0.899, 5.61]
	Vaccinated	1.39	[0.133, 15.2]
$c_{\inf}$ (/d)	Unvaccinated	0.565	[0.313, 2.64]
	Vaccinated	1.92	[0.307, 24.9]
$c_{\mathrm{RNA}}$ (/d)	Unvaccinated	0.160	[0.0881, 0.432]
	Vaccinated	0.398	[0.0635, 7.20]
ρ (copies/pfu)	Unvaccinated	1.88	[0.791, 2.65]
	Vaccinated	1.80	[0.175, 13.3]
$R_{0}$	Unvaccinated	13.8	[4.95, 18.3]
	Vaccinated	4.05	[1.45, 15.6]
$t_{\inf}$ (d)	Unvaccinated	0.681	[0.470, 0.764]
	Vaccinated	0.587	[0.229, 1.41]

**Table 2:** Average *p*-values for the Wilcoxon Rank-Sum and Levene tests. Values in bold indicate statistically significant results.

Parameter	Avg. *p*-Value (Wilcoxon)	Avg. *p*-Value (Levene)
β	0.059	**0.0091**
δ	0.40	0.36
*p*	0.56	0.15
$c_{\inf}$	**0.0039**	**0.030**
$c_{\mathrm{RNA}}$	**l0.0087**	**0.047**
ρ	0.44	**0.028**
$R_{0}$	**0.0015**	0.304
$t_{\inf}$	0.49	**0.0035**

## Data Availability

All data is included within the manuscript.
